# IMPACTS OF INTRATRIBAL DIVERSITY ON SERVICES FOR URBAN AMERICAN INDIAN ELDERS

**DOI:** 10.1093/geroni/igae098.1470

**Published:** 2024-12-31

**Authors:** Lyn Holley, Donna Polk

**Affiliations:** University of Nebraska at Omaha, Omaha, Nebraska, United States; Nebraska Urban Indian Health Coalition, Omaha, Nebraska, United States

## Abstract

This paper presents a report of analysis of interviews with members of the leadership team that serves Native American Indian elders in a mid-size American city. The city studied is home to members of more than a dozen different tribes; it has only one ethno-specific services center for older persons. While these older persons share a heritage of genocidal colonization, they are from different tribes and cultures. Native Americans residing in rural circumstances (e.g., reservations) are more likely to share and treasure similar beliefs and practices than are urban Indians. Diversity of beliefs and practices almost ineluctably brings occasional conflict - e.g., different beliefs about the place of women at the Drum or in singing, decorating for holidays, education of children, and suitability of different foods or ways of cooking. While literature on services for Native Americans is slowly growing, little notice has been taken of the challenges of serving this population in an urban setting. This paper begins to fill the gaps in the literature by describing the challenges and identifying methods that are useful for observation, description and analysis of cultural beliefs and practices of persons in the group, and for identifying which of these beliefs and practices support development of trust and cooperation, and which inhibit relationship building among Native American Indian Elders thus gaining and, importantly, applying knowledge of relevant cultures to inform design of activities and services for these Native Elders.

